# Supplementary material for: A Metabolomics Approach to Increasing Chinese Hamster Ovary (CHO) Cell Productivity
Source: Metabolites. 2021 Nov 30;11(12):823. doi: 10.3390/metabo11120823 (PMC8704136; doi:10.3390/metabo11120823)
Supplement: Supplementary file 1 [file metabolites-11-00823-s001.zip › metabolites-1403285-supplementary.pdf]

A Metabolomics Approach to Increasing CHO Cell Productivity
Supplementary Information

LC-MS Methods

Untargeted analysis was performed as described in Alden et al., 2017. In brief, information-dependent acquisition (IDA) experiments were performed on a QTOF, consisting of a TOF MS survey scan and four dependent product ion (MS/MS) scans for the highest intensity unique masses in each scan. Fragmentation was triggered when precursor ion counts rose quickly over several scans, ensuring ions were selected near the top of their LC peaks.

Reverse phase (RP) chromatography method

- Column: Phenomenex Synergi Hydro-RP
- Solvents:
  - A: 0.1% formic acid in water
  - B: 0.1% formic acid in methanol
- Column temperature: 15°C
- Flow rate: 0.2 mL/min
- Ion source: Turbo spray (ESI)
- Ion source Gas 1: 35
- Ion source Gas 2: 45
- Curtain Gas: 25
- Temperature: 450C
- IonSpray Voltage Floating: ±4500 V

Gradient:

**Time (min)**	**%B**
0-8	3
8-38	3 → 95
38-45	95
45-47	95 → 3
47-55	3

Hydrophilic interaction chromatography (HILIC) method

- Column: Phenomenex Luna NH2
- Solvents
  - A: 95:5 water:acetonitrile + 20mM ammonium acetate, pH to 9.45 using ammonium hydroxide
  - B: 100% acetonitrile
- Column temperature: 25°C
- Flow rate: 0.3 mL/min
- Ion source: Turbo spray (ESI)
- Ion source Gas 1: 35
- Ion source Gas 2: 45
- Curtain Gas: 25
- Temperature: 450C
- IonSpray Voltage Floating: ±5500 V

Gradient:

**Time (min)**	**%B**
0-15	85 → 0
15-28	0
28-30	0 → 85
30-60	85

For the QQQ targeted experiment, the HILIC method above was used with a modified gradient:

Gradient:

**Time (min)**	**%B**
0-15	85 → 0
15-20	0
20-25	0 → 85
25-30	85

Ion source: ESI
Source parameters
Gas temp: 350C
Gas flow: 11 l/min
Nebulizer: 50 psi
Capillary: Positive 4000V Negative 4500V

Table S1. MRM transitions and instrument parameters for targeted analysis

Compound Name	Mass	Precursor	Product	approx RT	CE	Frag	Polarity
4-aminobutanoate (GABA)	103.06	104.1	87.1	8.9	5	60	Positive
5-hydroxy-L-tryptophan	220.09	221.1	204.1	8.3	5	90	Positive
Adenosine	267.1	268.1	136.1	5.5	9	90	Positive
Biotin	244.09	245.1	227.1	9.7	5	90	Positive
Citrate	192.03	191	111.1	16.2	9	60	Negative
D-gluconic acid	196.16	195.2	75	11	17	90	Negative
D-glucuronate	194.04	193	73.1	11.6	9	90	Negative
Folate	441.14	440.1	310.9	17.3	21	147	Negative
Fumaric acid	116.01	115	71.1	16.1	5	60	Negative
Glycine	75.03	76	30.2	15	5	60	Positive
Guanine	151.05	152.1	135.1	7.1	17	120	Positive
Hypoxanthine	136.04	137	55.1	7.3	33	120	Positive
Inosine	268.08	269.1	137.1	5.2	5	60	Positive
L-2-aminoadipate	161.12	162.1	98.2	11.9	13	90	Positive
L-alanine	89.04	90.1	44.2	15	9	30	Positive
L-arginine	174.11	175.1	70.2	9.5	25	90	Positive
L-aspartate	133.04	132	88.1	11.7	9	60	Negative
L-cysteine	121.02	122	59.1	16	25	60	Positive
L-Cystine	240.02	239	120.1	11.9	5	60	Negative
L-Glutamate	147.05	148.1	84.1	11.7	13	60	Positive
L-histidine	155.07	156.1	110.1	9.1	9	90	Positive
L-isoleucine	131.1	132.1	86.2	6.6	5	60	Positive
L-leucine	131.1	132.1	86.2	6.6	5	60	Positive
L-lysine	146.1	147.1	84.2	10.2	13	90	Positive
L-methionine	149.05	148	47.1	7.4	9	60	Negative
L-Phenylalanine	165.08	166.1	120.1	7.1	9	60	Positive
L-proline	115.06	116.1	70.1	8	13	90	Positive
L-threonine	119.06	118	74.1	8.5	9	60	Negative
L-tryptophan	204.09	205.1	188.1	7.3	5	60	Positive
L-tyrosine	181.07	182.1	136.1	8.1	9	90	Positive
Methionine sulfoxide	165.04	166	74.1	8.9	9	90	Positive
S-malate	134.02	133	115.1	6.6	9	60	Negative
Succinate	118.03	117	73.1	15.5	9	60	Negative
Uridine	244.07	245.1	113	4.9	5	60	Positive

Table S2. Experimental design for add-back screening study

Exp. #	Clone	L-aspartic acid sodium salt monohydrate (mM)	γ-Aminobutyric acid (mM)	Sodium citrate (mM)	L-glutamic acid (mM)
1	B-6	0.72	20	3	0.76
2	B-1	0.72	5	3	0.76
3	B-6	0.18	5	12	0.19
4	B-1	0.72	20	12	0.19
5	A-5	0.36	10	6	0.38
6	A-5	0.72	5	3	0.76
7	B-1	0.36	10	6	0.38
8	B-1	0.18	20	3	0.18
9	A-2	0.72	5	12	0.76
10	A-2	0.72	5	3	0.76
11	A-2	0.36	10	6	0.38
12	B-6	0.18	5	3	0.76
13	B-1	0.72	20	12	0.19
14	B-6	0.72	5	12	0.76
15	A-2	0.72	20	12	0.19
16	A-5	0.36	10	6	0.38
17	A-5	0.72	20	3	0.76
18	A-2	0.18	20	12	0.76
19	A-5	0.72	5	3	0.19
20	A-2	0.72	20	12	0.19
21	B-6	0.72	20	12	0.76
22	A-2	0.72	5	3	0.76
23	A-2	0.36	10	6	0.38
24	A-2	0.18	5	12	0.19
25	A-2	0.72	20	3	0.76
26	B-6	0.18	5	3	0.76
27	B-6	0.36	10	6	0.38
28	B-1	0.72	5	3	0.76
29	B-1	0.18	5	12	0.19
30	B-6	0.72	5	3	0.19
31	A-2	0.72	5	12	0.76
32	A-5	0.18	5	12	0.19
33	B-1	0.72	20	3	0.76
34	B-1	0.72	20	12	0.76
35	B-1	0.18	20	12	0.76
36	B-6	0.18	20	3	0.19
37	B-6	0.18	20	3	0.19

Exp. #	Clone	L-aspartic acid sodium salt monohydrate (mM)	γ-Aminobutyric acid (mM)	Sodium citrate (mM)	L-glutamic acid (mM)
38	B-1	0.36	10	6	0.38
39	B-1	0.18	20	3	0.19
40	A-5	0.72	20	12	0.19
41	B-6	0.36	10	6	0.38
42	A-5	0.18	5	3	0.76
43	A-2	0.72	20	3	0.76
44	B-6	0.72	5	3	0.19
45	A-5	0.72	20	12	0.19
46	A-5	0.72	20	12	0.76
47	B-6	0.18	20	12	0.76
48	B-6	0.18	5	12	0.19
49	B-6	0.72	20	12	0.76
50	A-5	0.18	20	3	0.19
51	A-2	0.72	5	3	0.19
52	A-5	0.18	20	12	0.76
53	B-1	0.18	5	3	0.76
54	A-2	0.18	5	3	0.76
55	A-2	0.18	5	12	0.19
56	B-6	0.72	5	3	0.76
57	A-5	0.72	5	12	0.76
58	B-1	0.18	5	12	0.19
59	A-5	0.18	20	12	0.76
60	B-6	0.72	20	12	0.19
61	B-1	0.72	5	3	0.19
62	A-5	0.72	5	3	0.19
63	B-6	0.18	20	12	0.76
64	A-2	0.18	20	12	0.76
65	A-5	0.72	5	12	0.76
66	A-2	0.72	20	12	0.76
67	B-1	0.18	5	3	0.76
68	B-6	0.72	20	12	0.18
69	B-1	0.72	5	12	0.76
70	A-2	0.18	20	3	0.19
71	B-1	0.72	5	3	0.19
72	B-1	0.72	20	12	0.76
73	A-5	0.72	5	3	0.76
74	A-5	0.18	20	3	0.19
75	B-6	0.72	5	3	0.76
76	A-5	0.72	20	3	0.76

Exp. #	Clone	L-aspartic acid sodium salt monohydrate (mM)	γ-Aminobutyric acid (mM)	Sodium citrate (mM)	L-glutamic acid (mM)
77	A-2	0.72	20	12	0.76
78	A-2	0.18	20	3	0.19
79	B-1	0.72	5	12	0.76
80	B-1	0.72	20	3	0.76
81	A-5	0.72	20	12	0.76
82	A-2	0.72	5	3	0.19
83	A-5	0.18	5	3	0.76
84	A-2	0.18	5	3	0.76
85	A-5	0.18	5	12	0.19
86	B-6	0.72	5	12	0.76
87	B-6	0.72	20	3	0.76
88	B-1	0.18	20	12	0.76
89	B-1	0	0	0	0
90	B-1	0	0	0	0
91	A-2	0	0	0	0
92	A-2	0	0	0	0
93	B-6	0	0	0	0
94	B-6	0	0	0	0
95	A-5	0	0	0	0
96	A-5	0	0	0	0

A D-optimal design of 96 conditions was used to screen four metabolites for qP-enhancing potential.

Table S3. Pearson correlation coefficients between annotated metabolites and qP or growth.

	Correlation with qP		Correlation with growth	
**Annotated metabolite**	**Correlation coefficient**	**p-value**	**Correlation coefficient**	**p-value**
L-Glutamate 5-semialdehyde	0.817	0.008	-0.829	0.030
2-Oxobutanoate	0.799	0.010	-0.827	0.030
2-Hydroxy-dATP	0.901	0.002	-0.738	0.032
Biotin	0.875	0.004	-0.692	0.032
cis-Aconitate	0.815	0.008	-0.696	0.032
Citrate	0.822	0.008	-0.700	0.032
Biotin amide	0.813	0.008	-0.732	0.032
D-Glucuronate	0.806	0.009	-0.734	0.032
5-Hydroxyindoleacetaldehyde	0.787	0.011	-0.739	0.032
L-Formylkynurenine	0.782	0.011	-0.803	0.032
N-Acetyl-D-mannosamine	0.787	0.011	-0.740	0.032
N-Acetylneuraminate	0.772	0.013	-0.736	0.032
Adenosine	0.766	0.014	-0.708	0.032
L-Tryptophan	0.762	0.014	-0.732	0.032
L-Arginine	0.758	0.015	-0.756	0.032
5-Hydroxy-L-tryptophan	0.755	0.015	-0.707	0.032
alpha-D-Glucose	0.740	0.019	-0.776	0.032
Guanine	0.732	0.021	-0.702	0.032
7,8-Dihydroneopterin 3'-triphosphate	0.722	0.022	-0.700	0.032
Inosine	0.722	0.022	-0.724	0.032
L-Histidine	0.697	0.028	-0.721	0.032
L-Phenylalanine	0.697	0.028	-0.715	0.032
L-Isoleucine	0.691	0.029	-0.780	0.032
L-Methionine	0.683	0.031	-0.776	0.032
5-Acetylamino-6-formylamino-3-methyluracil	0.667	0.038	-0.717	0.032
L-methionine-S-oxide	0.658	0.041	-0.767	0.032
Sorbitol 6-phosphate	0.649	0.044	-0.810	0.032
Leukotriene C4	0.639	0.046	-0.695	0.032
Sucrose	0.635	0.048	-0.775	0.032
Glycine	0.839	0.007	-0.684	0.034
L-2-Aminoadipate	0.784	0.011	-0.684	0.034
Folate	0.781	0.011	-0.683	0.034
Dihydrobiopterin	0.751	0.016	-0.675	0.036
L-Glutamate	0.907	0.002	-0.665	0.038
Succinate	0.836	0.007	-0.656	0.041
5-Oxoproline	0.918	0.002	-0.638	0.049
4-Aminobutanoate	0.897	0.002	-0.626	0.053
Farnesylcysteine	0.865	0.005	-0.626	0.053

L-Alanine	0.722	0.022	-0.627	0.053
sn-Glycero-3-phospho-1-inositol	0.818	0.008	-0.621	0.054
5,10-Methenyltetrahydrofolate	0.700	0.027	-0.623	0.054
UDP-N-acetyl-alpha-D-glucosamine	0.701	0.027	-0.611	0.058
Uridine	0.780	0.011	-0.582	0.072
Glycolate	0.653	0.042	-0.582	0.072
(R)-Mevalonate	0.779	0.011	-0.567	0.079
L-Aspartate	0.760	0.015	-0.548	0.088
(R)-5-Phosphomevalonate	0.914	0.002	-0.531	0.097
Fumarate	0.756	0.015	-0.524	0.102
(S)-1-Pyrroline-5-carboxylate	0.802	0.009	-0.509	0.113
sn-Glycero-3-phosphoethanolamine	0.640	0.046	-0.507	0.113
D-Gluconic acid	0.810	0.009	-0.489	0.128
2-Hydroxy-dAMP	0.817	0.008	-0.430	0.182
(S)-Malate	0.770	0.013	-0.410	0.205
Hypoxanthine	0.643	0.045	-0.372	0.249
3-Cyano-L-alanine	0.618	0.056	-0.696	0.032
3-Aminopropanal	0.614	0.057	-0.741	0.032
2,5-Dioxopentanoate	0.569	0.085	-0.711	0.032
L-Proline	0.558	0.092	-0.704	0.032
L-Tyrosine	0.536	0.105	-0.782	0.032
D-Sorbitol	0.524	0.116	-0.780	0.032
L-Threonine	0.506	0.132	-0.696	0.032
1-(5-Phospho-D-ribosyl)-5-amino-4-imidazolecarboxylate	0.460	0.178	-0.775	0.032
L-Lysine	0.458	0.178	-0.701	0.032
L-Leucine	0.443	0.191	-0.702	0.032
Carboxyphosphamide	0.406	0.234	-0.750	0.032
S-Adenosyl-L-homocysteine	0.401	0.240	-0.700	0.032
4-Fumarylacetoacetate	0.391	0.252	-0.761	0.032
Dopamine	0.305	0.375	-0.713	0.032
L-Cysteine	0.327	0.344	-0.676	0.036
D-myo-Inositol 1,2-cyclic phosphate	0.322	0.351	-0.674	0.036
2-Aminobut-2-enoate	0.549	0.098	-0.665	0.038
5-Guanidino-2-oxopentanoate	0.426	0.211	-0.667	0.038
L-Cystine	0.387	0.257	-0.663	0.038
Dopaquinone	0.627	0.052	-0.652	0.042
Betaine	0.358	0.296	-0.618	0.055
Pterine	0.411	0.229	-0.568	0.079

6-Phospho-D-gluconate	0.200	0.575	-0.560	0.081
L-Glutamine	0.167	0.637	-0.558	0.082
L-Pipecolate	0.617	0.056	-0.545	0.089
Taurine	0.469	0.169	-0.536	0.094
L-Asparagine	0.180	0.613	-0.513	0.109
Guanidinoacetate	0.365	0.286	-0.455	0.158
5,10-Methylenetetrahydrofolate	0.503	0.134	-0.435	0.178
L-Serine	-0.188	0.598	-0.405	0.209
D-Glycerate	0.241	0.492	-0.401	0.213
(S)-3-Methyl-2-oxopentanoic acid	0.008	0.986	-0.396	0.218
1,2-Dibromoethane	0.540	0.103	-0.376	0.245
L-Cysteate	0.424	0.211	-0.368	0.252
3-Methoxy-4-hydroxyphenylglycolaldehyde	0.581	0.077	-0.326	0.314
3,4-Dihydroxymandelate	0.313	0.366	-0.293	0.368
1D-myo-Inositol 1,3,4,5,6-pentakisphosphate	-0.044	0.915	0.270	0.406
Alcophosphamide	-0.302	0.378	-0.119	0.717

Table S4. Metabolites reaching at least MSI level 2 identification.

dataSrc	mz	RT	ppm	Confirmed with standard	Annotation	Match Factor
HilNeg	74.02558	618.8435	11.40		Glycine	597
HilNeg	105.0188	759.329	4.68		Glyceric acid	917
HilNeg	114.0546	574.902	12.58	X	L-Proline	661
HilNeg	118.05	610.716	7.92		Threonine	668
HilNeg	145.0613	616.1975	3.70	X	L-Glutamine	947
HilNeg	164.0372	639.561	9.39	X	L-methionine S-Oxide	761
HilNeg	195.0502	800.8455	4.01		D-gluconic acid	782
HilNeg	239.016	853.677	2.12		L-Cystine	938
HilNeg	440.1362	1229.261	8.49	X	Folic acid	899
HilPos	77.02329	503.784	0.36		Glycolic acid	700
HilPos	106.0484	636.0098	13.71	X	Serine	876
HilPos	130.0864	673.7855	1.05		Pipecolic acid	729
HilPos	133.0606	631.9407	1.19	X	L-Asparagine	976
HilPos	137.0451	411.3256	5.14	X	Hypoxanthine	911
HilPos	152.0564	527.1319	1.81		Guanine	523
HilPos	156.0764	646.861	2.24	X	L-Histidine	926
HilPos	175.1187	636.9453	1.52		L-Arginine	990
HilPos	176.0728	582.9782	12.74		5-hydroxyindoleacetaldehyde	*
HilPos	221.0889	519.4064	14.41		5-hydroxy-l-tryptophan	684
SynNeg	88.04049	881.1325	1.02	X	L-alanine	587
SynNeg	101.0244	920.672	0.02	X	2-oxobutanoate	659
SynNeg	112.0399	1289.605	5.04		(S)-1-Pyrroline-5-carboxylate	504
SynNeg	115.003	882.8115	5.77		Fumaric acid	582
SynNeg	117.019	2587.291	3.00	X	Succinate	887

SynNeg	132.03	883.0335	1.89	X	L-aspartic acid	988
SynNeg	133.0136	1382.16	4.67	X	Malic acid	987
SynNeg	145.0975	717.552	4.88	X	L-Lysine	719
SynNeg	146.0455	910.85	2.49	X	L-Glutamic acid	752
SynNeg	148.0433	1443.241	3.48	X	L-Methionine	636
SynNeg	160.0607	1154.211	5.31	X	L-2-aminoadipic acid	806
SynNeg	162.0414	2615.492	4.31		Pterine	798
SynNeg	164.0717	2816.974	0.18	X	L-phenylalanine	903
SynNeg	173.0082	2452.754	5.06		cis-aconitic acid	572
SynNeg	180.0666	2490.638	0.33	X	L-tyrosine	922
SynNeg	191.0193	2453.592	2.28	X	Citric acid	970
SynNeg	193.0348	928.936	2.80		D-glucuronic acid	965
SynNeg	203.0826	2984.451	0.06	X	L-tryptophan	962
SynNeg	346.0572	2337.519	3.92		Adenosine monophosphate	815
SynPos	115.0496	2885.132	5.10		3-Cyano-L-alanine	483
SynPos	245.092	3140.981	13.97	X	Biotin	905

*No score but see Alden et al, *Metabolites* 2020, *10*(5), 199; https://doi.org/10.3390/metabo10050199

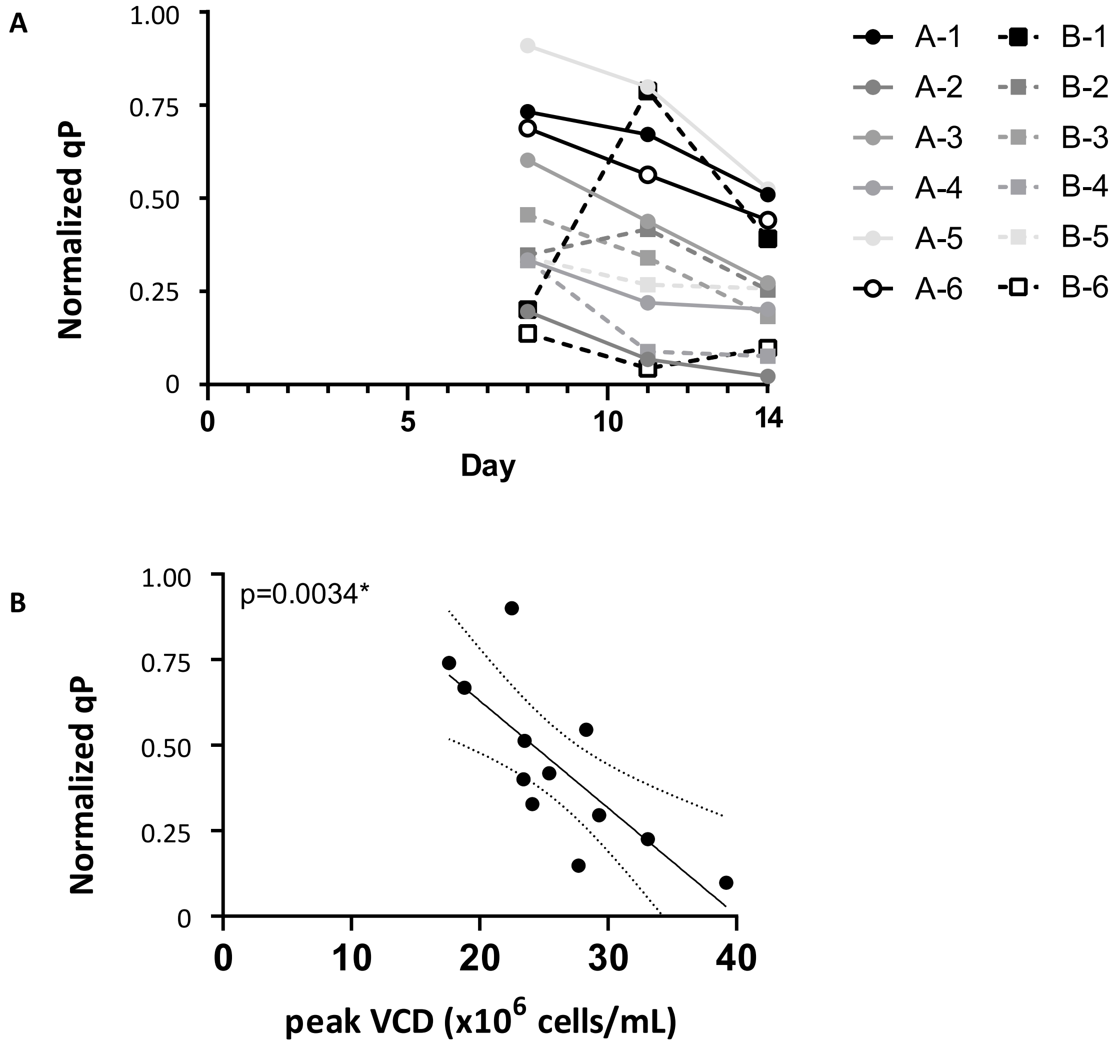

Figure S1. Time profiles of qP for twelve clones producing mAb A and mAb B. (A) For the overall comparison of qP in Figure 1C, titers from days 6 and 11 were used. More detailed qP profiles for each clone are shown here, using titer data available on days 6, 8, 11, and 14. (B) Peak VCD was negatively correlated with qP (days 6-11), indicating a tradeoff between productivity and growth.

Figure S2. Mirror plot examples. The red spectra are from samples; the blue spectra are from standards. Spectral scores (head to tail match factors and reverse match factors) in green indicate the similarity of the unknown spectrum to the library spectrum on the NIST scale of 0 (no matching peaks) to 999 (perfect match).

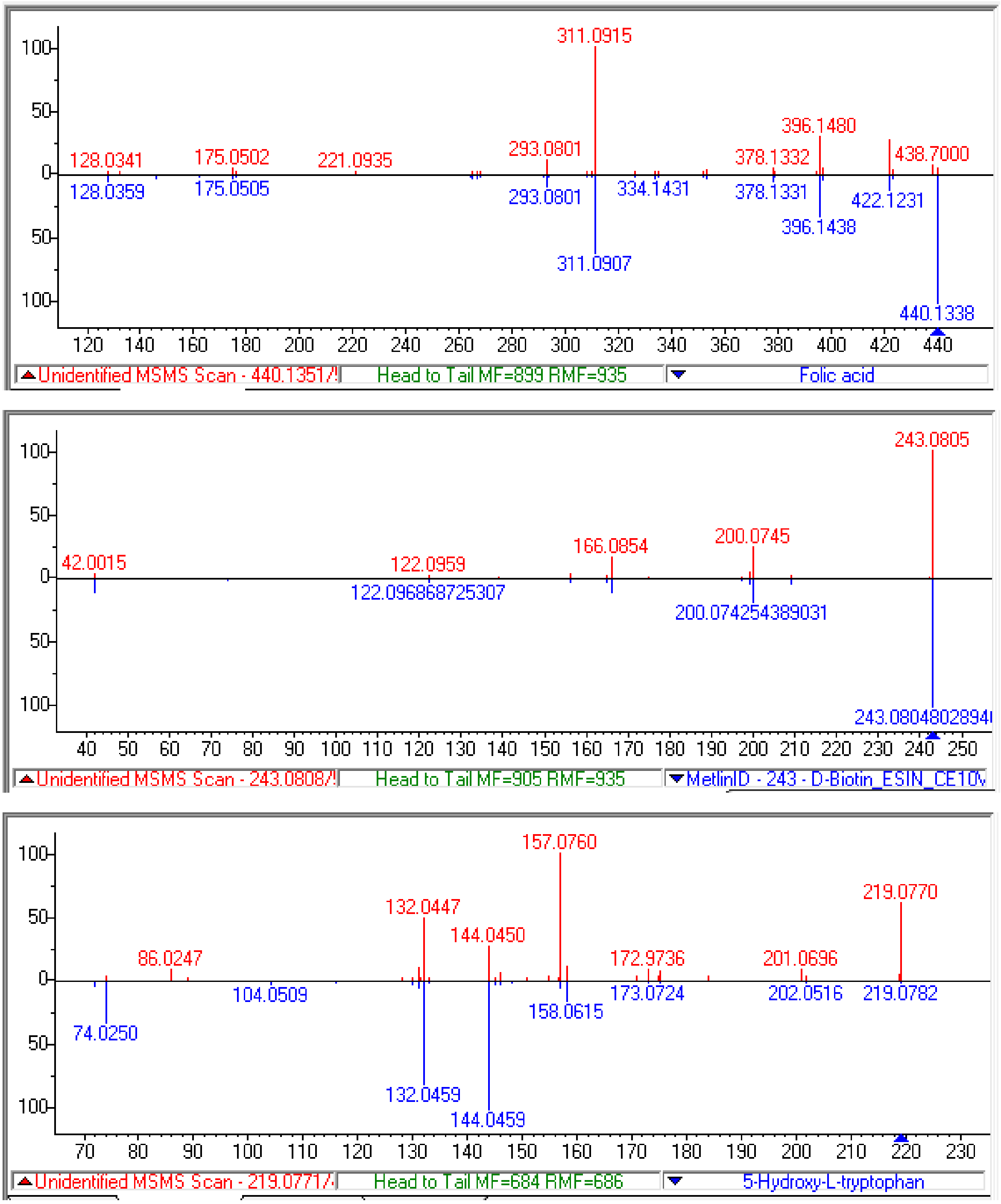

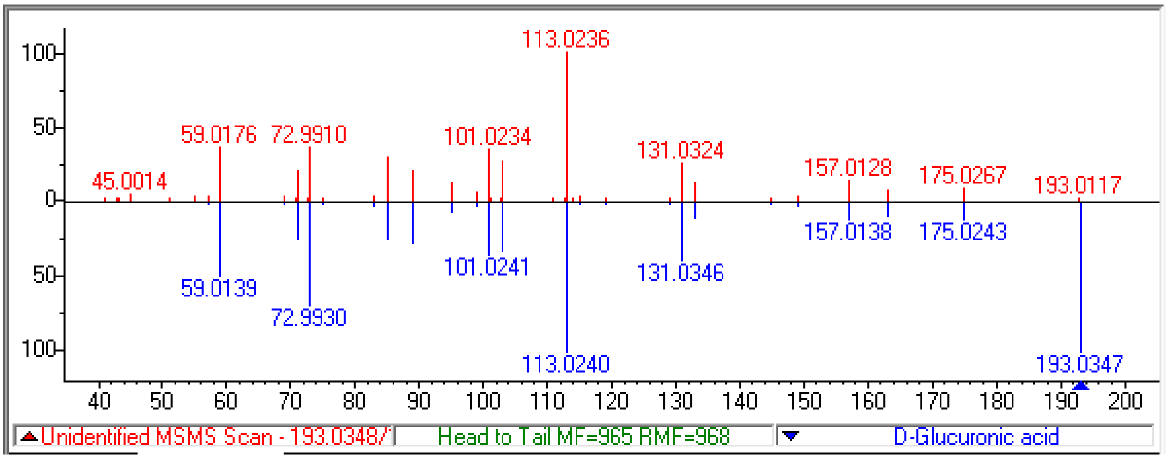

113.0236
59.0176
72.9910
101.0234
131.0324
157.0128
175.0267
193.0117
45.0014
59.0139
72.9930
101.0241
131.0346
157.0138
175.0243
113.0240
193.0347
Unidentified MSMS Scan - 193.0348/
Head to Tail MF=965 RMF=968
D-Glucuronic acid

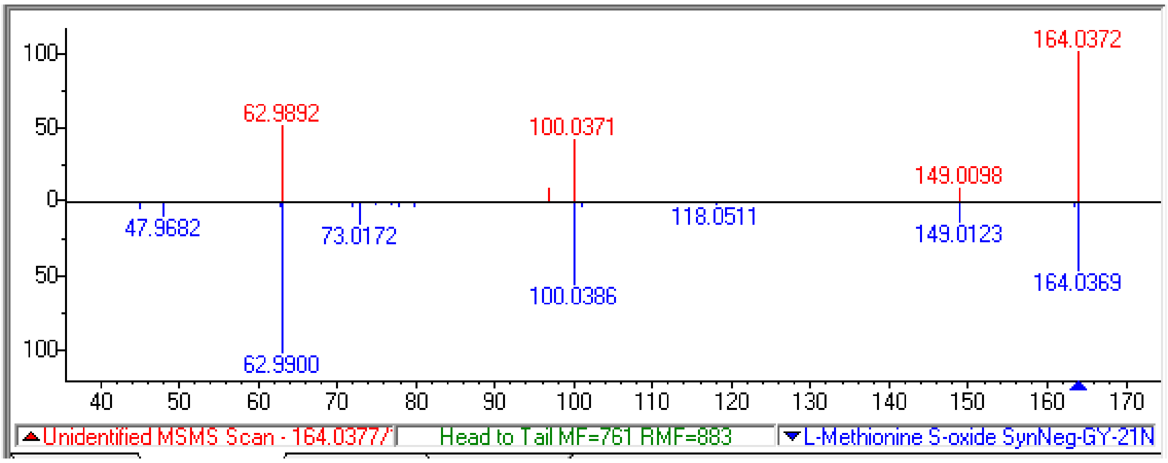

164.0372
62.9892
100.0371
149.0098
47.9682
73.0172
118.0511
149.0123
164.0369
100.0386
62.9900
Unidentified MSMS Scan - 164.0377/
Head to Tail MF=761 RMF=883
L-Methionine S-oxide SynNeg-GY-21N

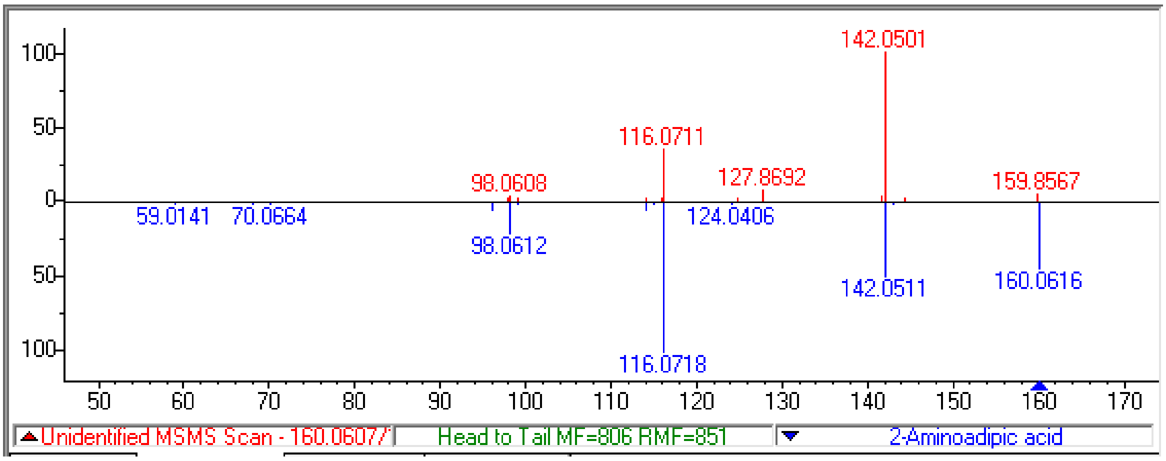

142.0501
116.0711
98.0608
127.8692
159.8567
59.0141
70.0664
124.0406
98.0612
142.0511
160.0616
116.0718
Unidentified MSMS Scan - 160.0607/
Head to Tail MF=806 RMF=851
2-Aminoadipic acid

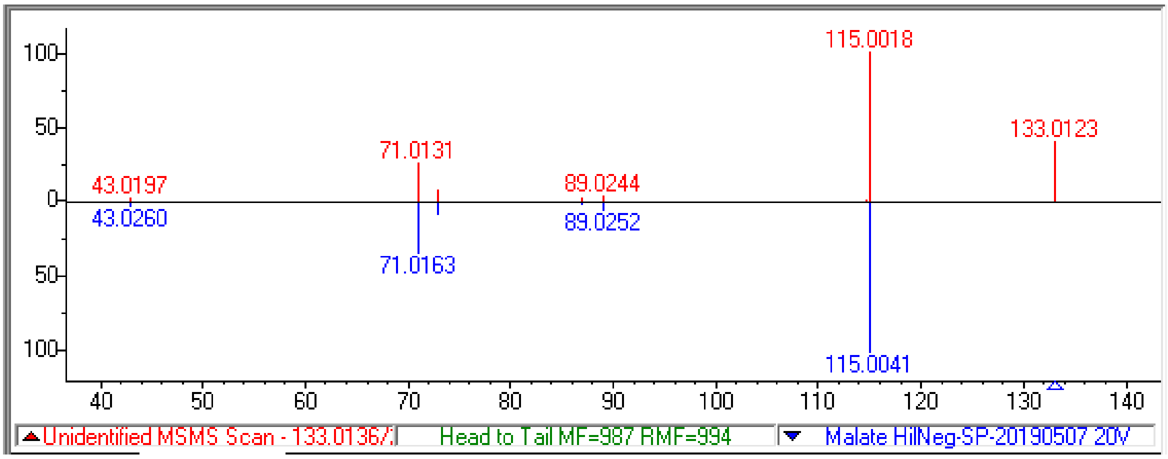

115.0018
133.0123
71.0131
43.0197
89.0244
43.0260
89.0252
71.0163
115.0041
Unidentified MSMS Scan - 133.01367
Head to Tail MF=987 RMF=994
Malate HilNeg-SP-20190507 20V

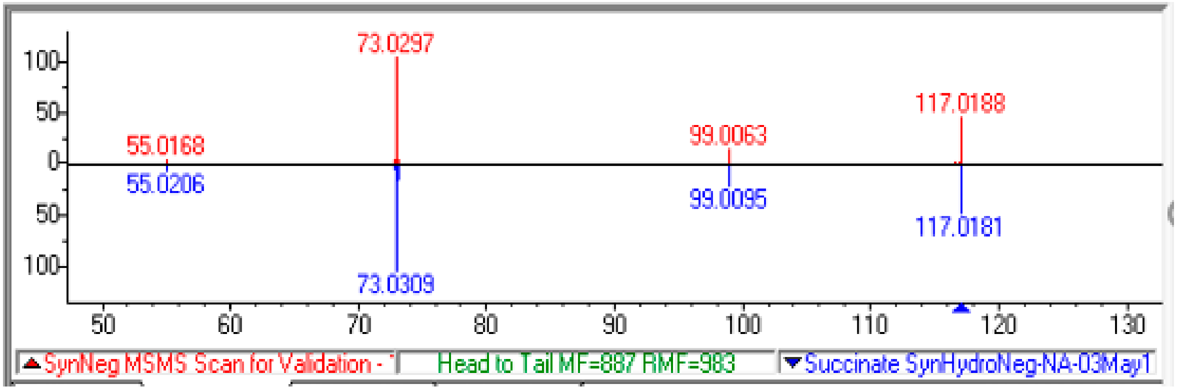

73.0297
117.0188
99.0063
55.0168
55.0206
99.0095
117.0181
73.0309
SynNeg MSMS Scan for Validation -
Head to Tail MF=887 RMF=983
Succinate SynHydroNeg-NA-03May1

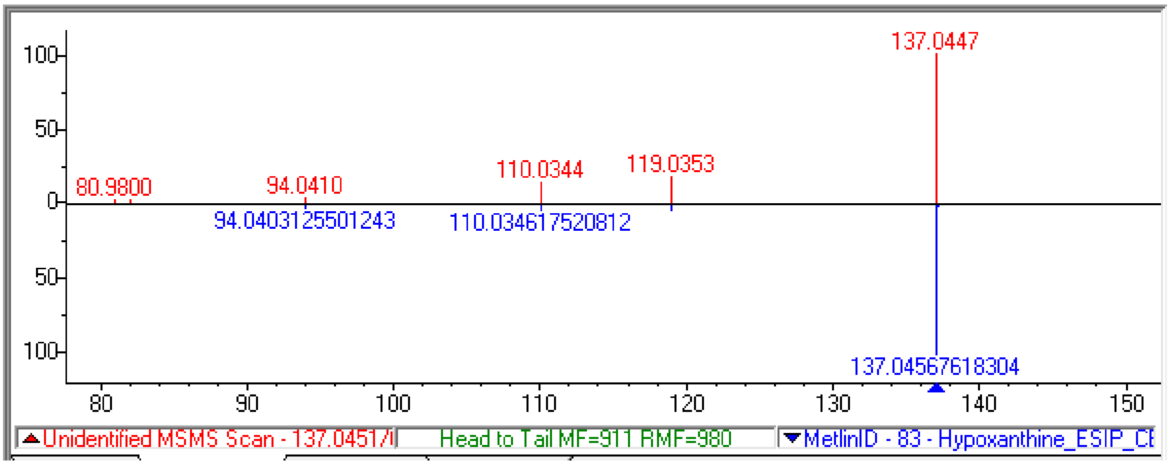

137.0447
110.0344
119.0353
80.9800
94.0410
94.0403125501243
110.034617520812
137.04567618304
Unidentified MSMS Scan - 137.0451
Head to Tail MF=911 RMF=980
MetlinID - 83 - Hypoxanthine_ESIP_CE

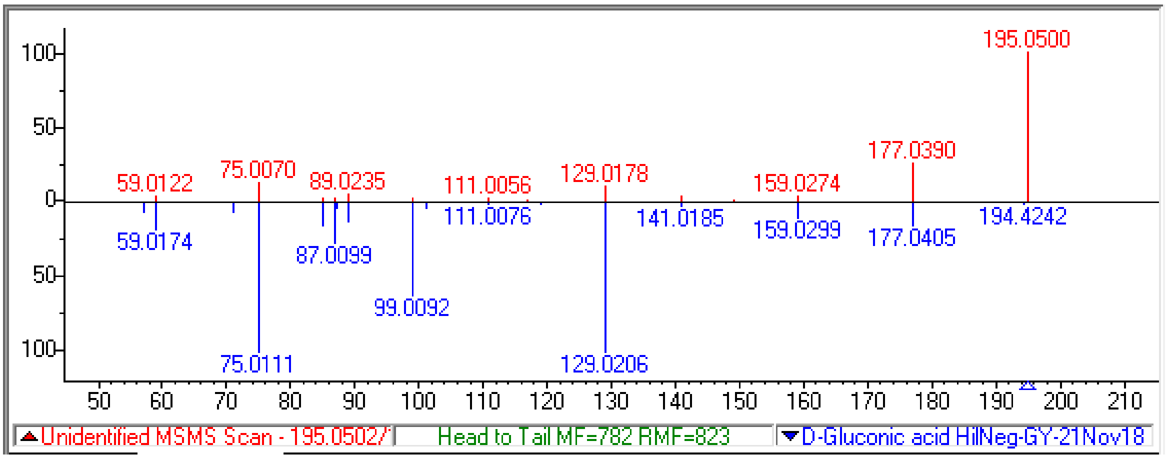

195.0500
177.0390
159.0274
129.0178
111.0056
89.0235
75.0070
59.0122
59.0174
75.0111
87.0099
99.0092
111.0076
129.0206
141.0185
159.0299
177.0405
194.4242
Unidentified MSMS Scan - 195.0502/
Head to Tail MF=782 RMF=823
D-Gluconic acid HilNeg-GY-21Nov18

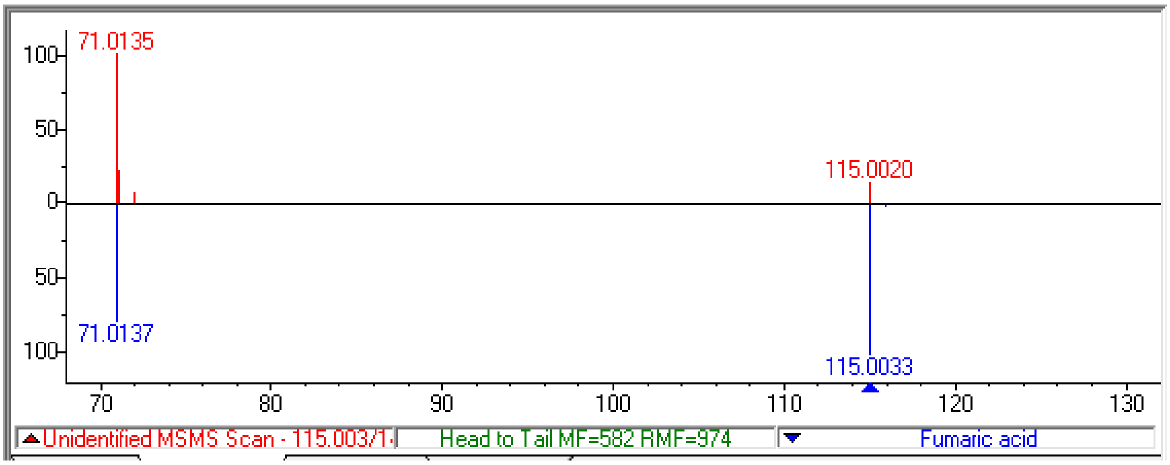

71.0135
115.0020
71.0137
115.0033
Unidentified MSMS Scan - 115.003/1
Head to Tail MF=582 RMF=974
Fumaric acid

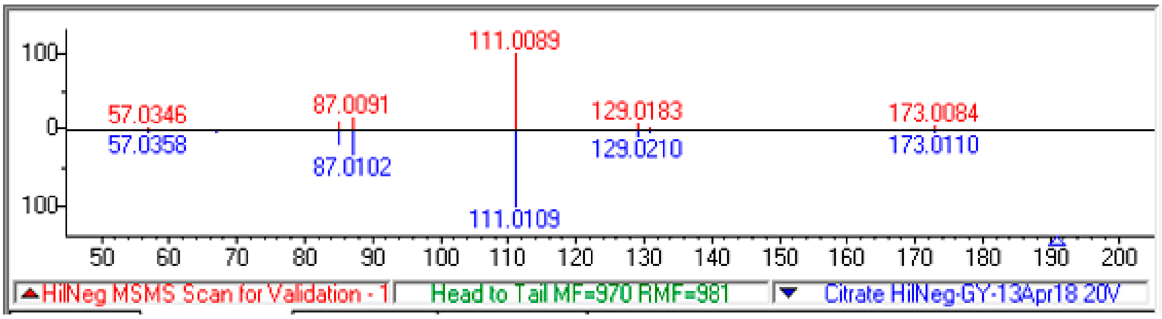

111.0089
57.0346
87.0091
129.0183
173.0084
57.0358
87.0102
129.0210
173.0110
111.0109
HilNeg MSMS Scan for Validation - 1
Head to Tail MF=970 RMF=981
Citrate HilNeg-GY-13Apr18 20V

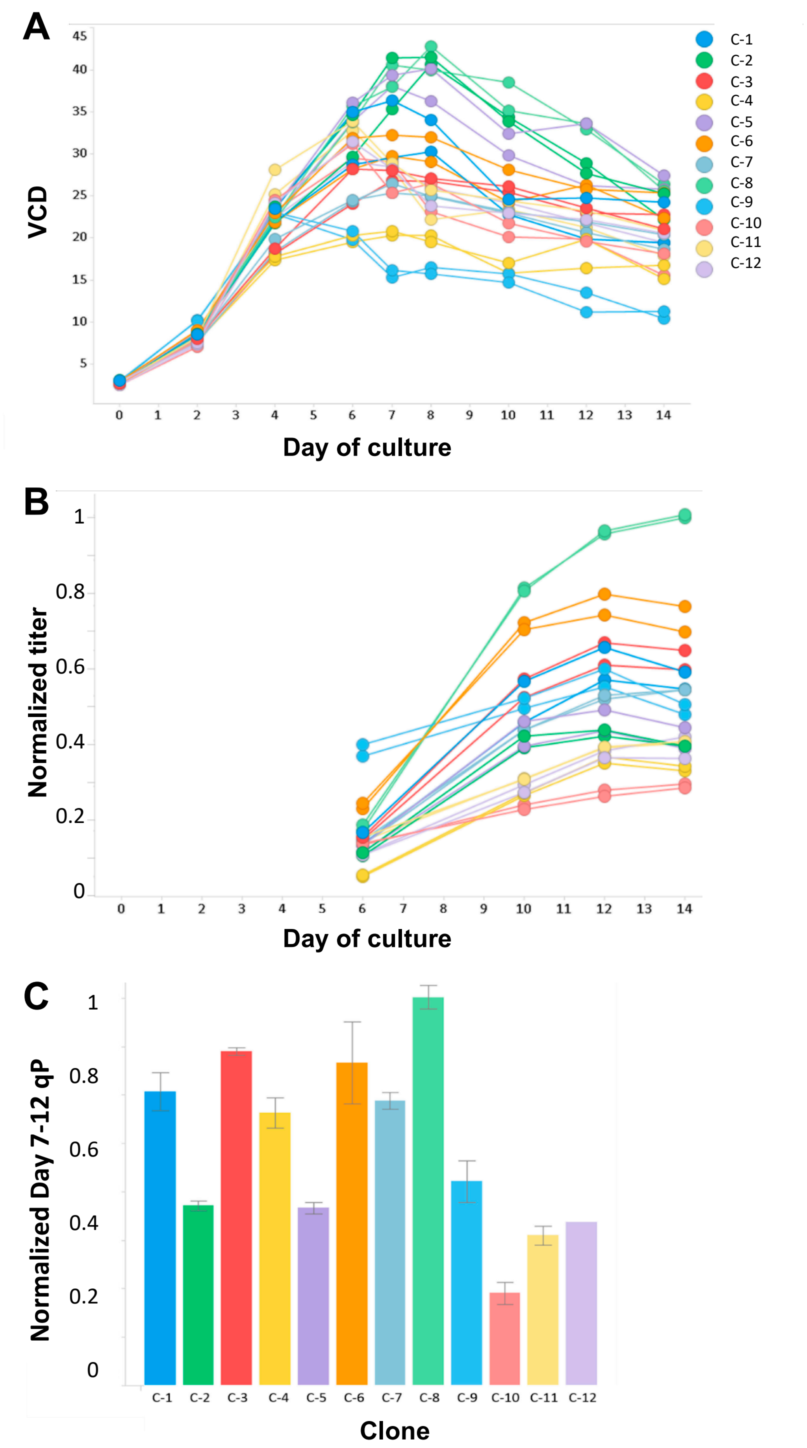

Figure S3. Growth and productivity profiles of mAb C experiment. A set of 12 clones expressing mAb C was cultured in duplicate in Ambr 15 bioreactors using the same fed-batch process as the 5-L bioreactor cultures used for the first set of 12 clones expressing mAb A or mAb B. (A) Growth and (B) productivity profiles. (C) Average qP was calculated for correlation analysis from Day 7 and Day 12 titers. Error bars indicate standard deviation between duplicates.

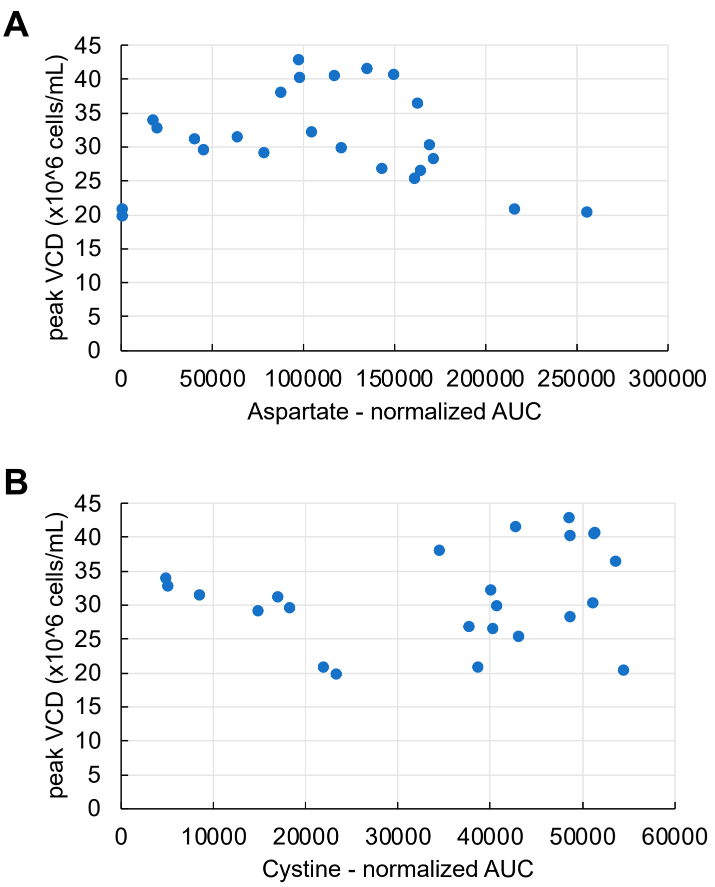

Figure S4. Correlations between Day 7 metabolite levels and peak VCD. (**A**) Aspartate and (**B**) cystine AUCs in the targeted experiment were normalized to Day 7 VCDs and plotted against peak VCD. Calculations of both Pearson and Spearman's rank correlation coefficients showed that there were no significant correlations between the metabolite levels and peak VCD.

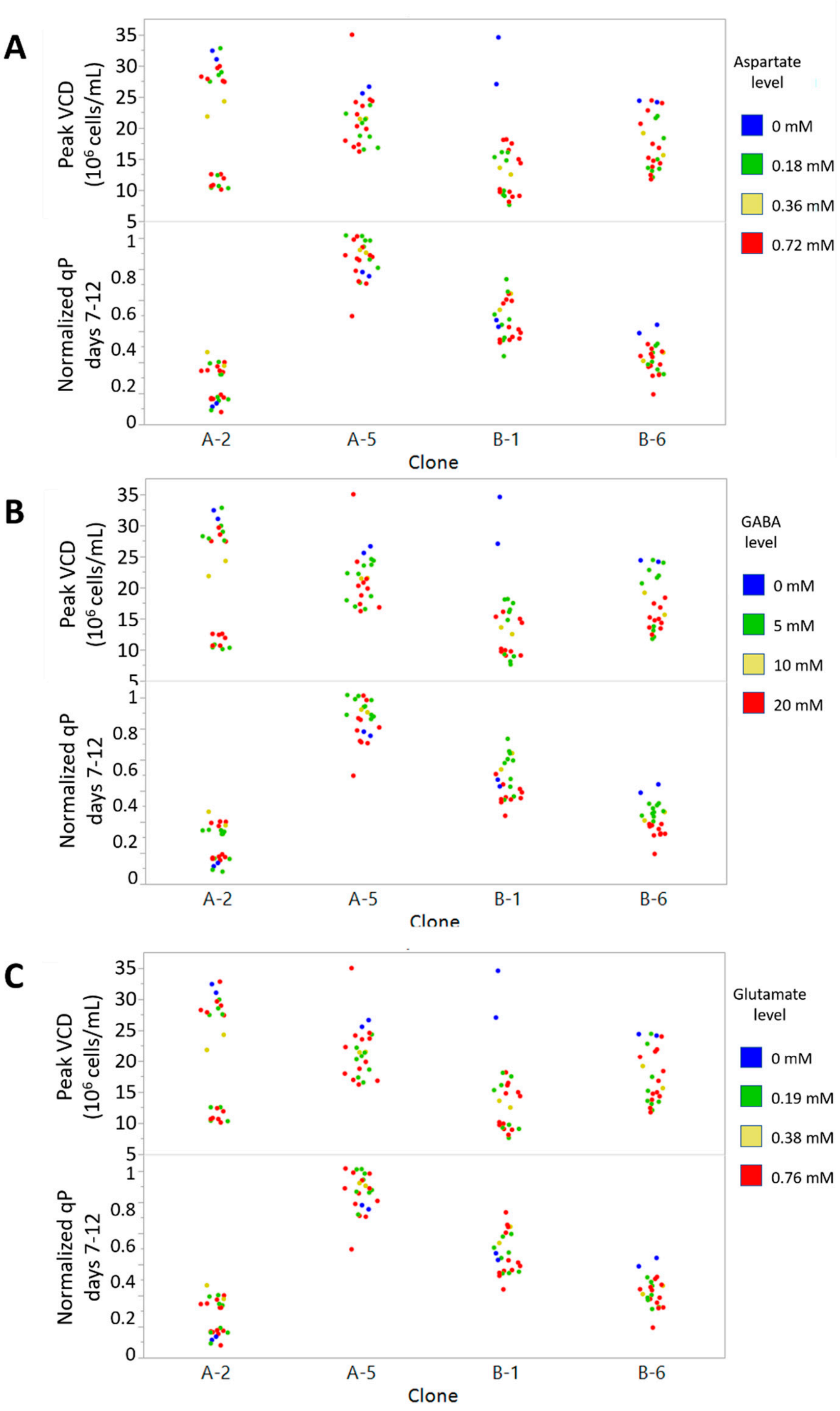

Figure S5. Productivity and growth in the add-back screening study. Data shown are cell-specific productivity (qP) and peak VCD of the 96 conditions (Table S2). (A) Aspartate, (B) GABA, (C) glutamate. Colors indicate metabolite level. Value 0 represents the control and 3 represents the highest level of the indicated metabolite. In contrast to citrate (Figure 4), these metabolites did not show consistent responses in qP or VCD. High-qP clones used in this study were A-5 and B-1. Low-qP clones were A-2 and B-6.

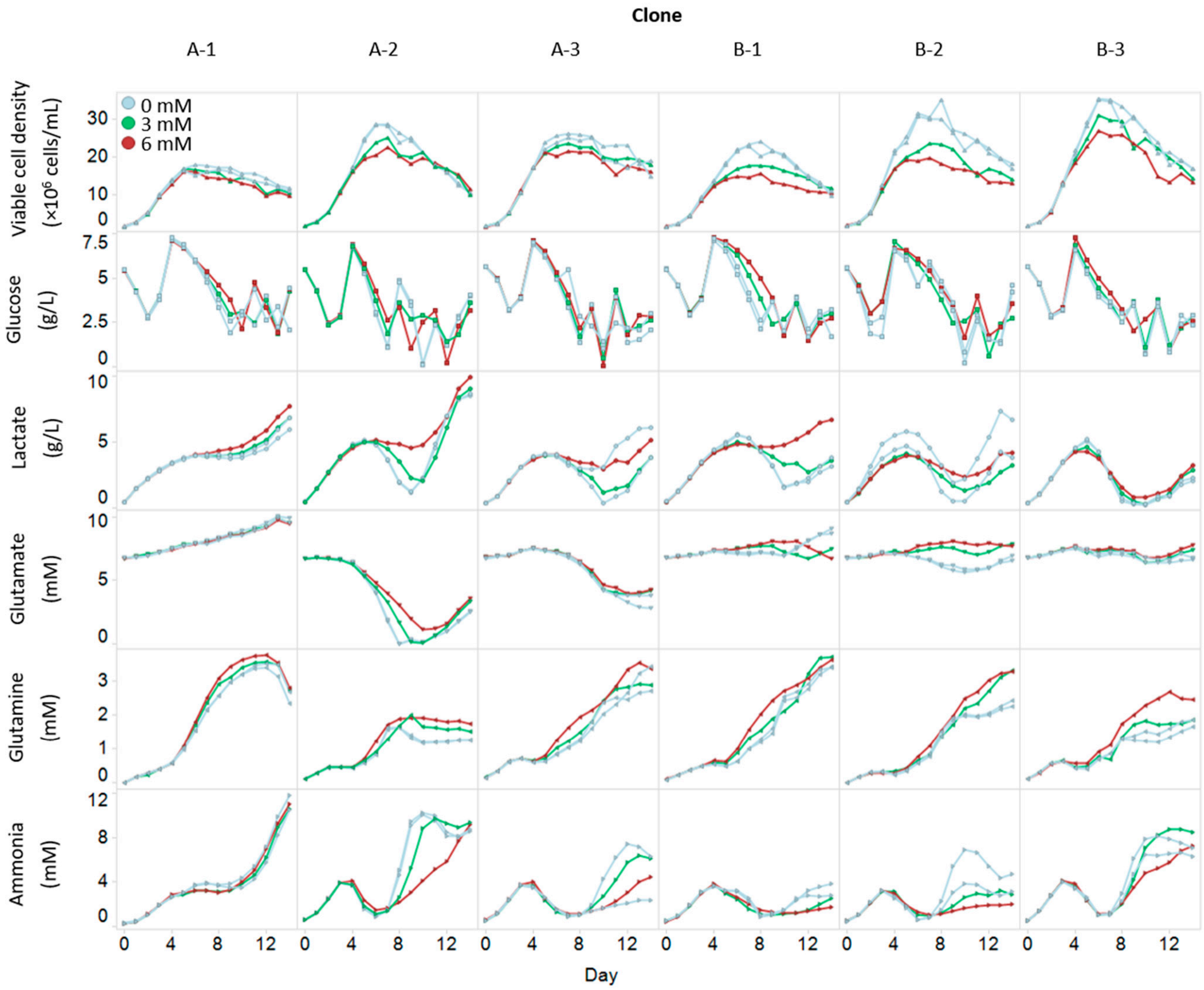

Figure S6. Metabolite profiles for citrate add-back study in Ambr 250 reactors. Line colors represent different amounts of citrate supplementation. The control condition was performed in duplicate. For all but one clone (A-1), we observed a dose-dependent decrease in peak VCD, an increase in lactate accumulation, and a decrease in lactate accumulation with the addition of citrate. We also saw slightly increased glutamate and glutamine concentrations.

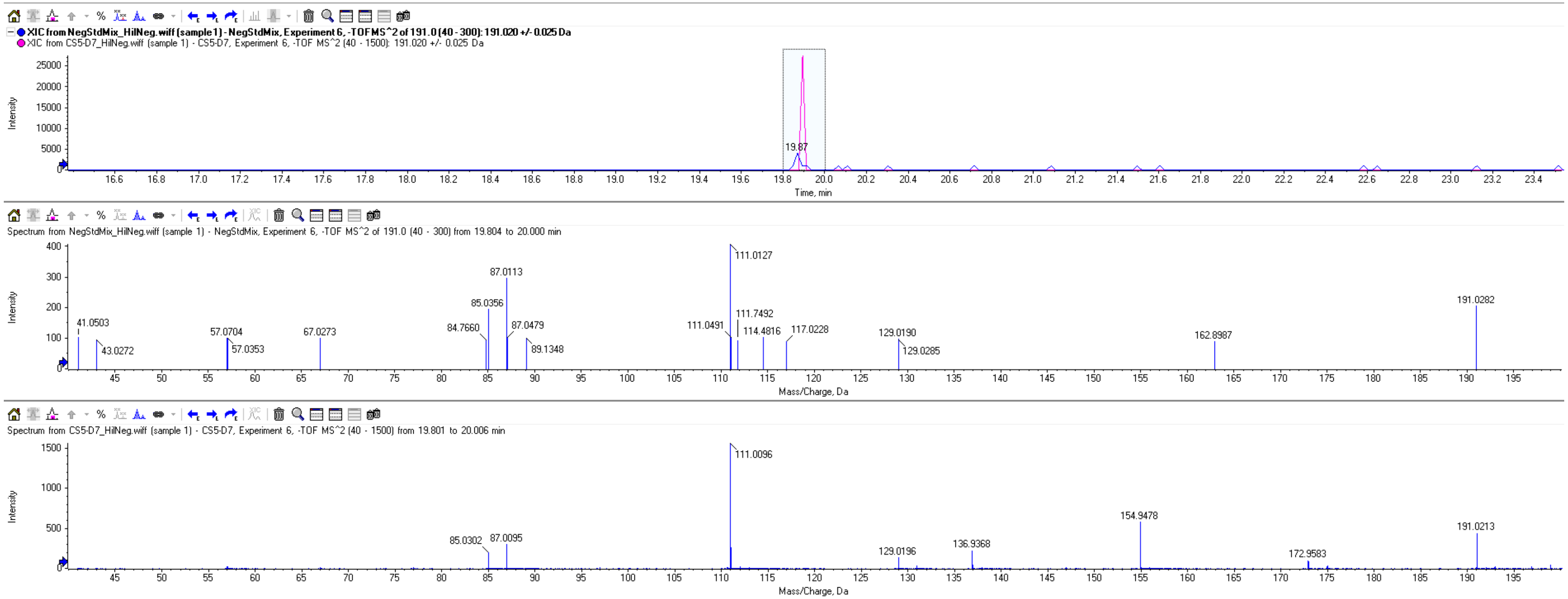

Figure S7. Example chromatogram from untargeted LC-MS analysis. The top panel is the XIC (extracted ion chromatogram) showing the retention time for citrate when run in the negative ionization mode using the HILIC column. The next panels are the MS/MS spectra from the selected XIC window for a mix of standards including citrate (center panel) and a sample (bottom panel).

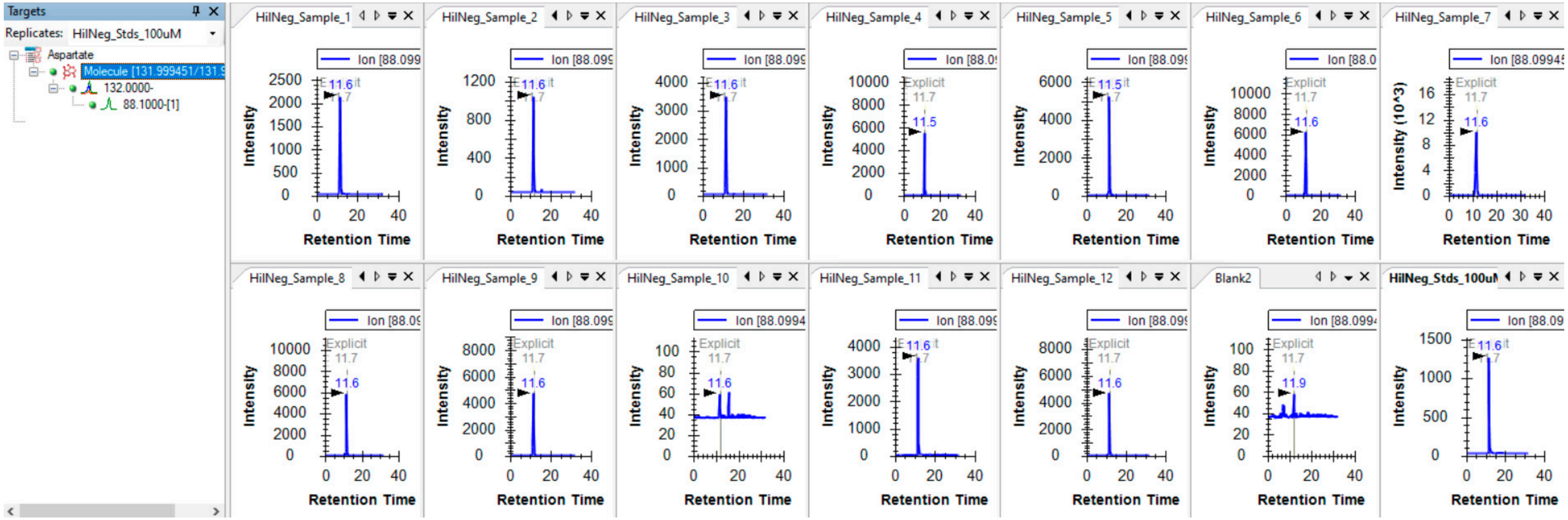

Figure S8. Example chromatograms from targeted LC-MS analysis. Varying intensities can be seen for aspartate in supernatant samples. The last two panels show a very low intensity in a blank sample and a higher intensity for a chemical standard.
